# Enduring insights from sex differences in COVID-19: a retrospective *post hoc* analysis from a large Italian patient cohort

**DOI:** 10.3389/fmed.2026.1764966

**Published:** 2026-02-11

**Authors:** Carmine Siniscalchi, Angela Guerra, Nicoletta Cerundolo, Pierpaolo Di Micco, Claudio Tana, Beatrice Prati, Alberto Parise, Antonio Nouvenne, Tiziana Meschi

**Affiliations:** 1 Department of Internal Medicine, Parma University Hospital, Parma, Italy; 2 Internal Medicine Ward “P.O. Santa Maria delle Grazie” Pozzuoli, Naples, Italy; 3 Internal Medicine Unit, Eastern Hospital, Asl Taranto, Italy

**Keywords:** COVID-19, frailty, inflammatory biomarkers, non-vaccinated population, radiological severity, SARS-CoV-2, sex differences

## Abstract

**Background:**

In the early phase of the coronavirus disease 2019 (COVID-19) pandemic, before containment strategies and without vaccines or targeted therapies, clinical outcomes among hospitalized patients were heterogeneous. Preliminary reports suggested possible sex-related differences in susceptibility and disease severity. This study examined sex-based differences in clinical presentation, radiological involvement, laboratory findings, and in-hospital outcomes among patients admitted during the pre-lockdown phase of the outbreak in Italy.

**Methods:**

We conducted a retrospective observational study of 689 consecutive adults hospitalized at Parma University Hospital between 28 February and 22 March 2020, with chest CT findings consistent with COVID-19–related interstitial pneumonia, regardless of RT-PCR results. Demographics, comorbidities, symptoms, laboratory parameters, and outcomes were compared between males and females. Lung involvement was quantified using a CT visual score. Multivariable logistic regression identified predictors of in-hospital mortality.

**Results:**

Females accounted for 39% of the cohort and were significantly older and frailer than males, with a higher prevalence of Rockwood ≥7 (15 vs. 5%). Despite this, females demonstrated a lower CT visual score (25 vs. 30%), higher oxygenation parameters, and a less pronounced inflammatory and tissue-damage profile, including lower CRP, LDH, and CPK. Females had shorter hospital stays (5 vs. 6 days) and lower age-adjusted mortality. Multivariable analysis confirmed female sex as independently protective (OR 0.597), an effect largely mediated by reduced radiological lung involvement and attenuated inflammatory response.

**Conclusions:**

Females showed milder radiological and biochemical profiles and reduced mortality despite being older and frailer. These findings highlight intrinsic sex-related biological differences in host response. These early patterns remain relevant today, as sex-specific vulnerability may inform precision medicine and risk stratification.

## Introduction

Since the emergence of coronavirus disease 2019 (COVID-19), substantial heterogeneity in clinical presentation and outcomes has been observed among affected individuals. Early epidemiological reports consistently indicated that males were disproportionately affected by severe disease and mortality compared with females (1–3). However, most available data were generated during phases of the pandemic in which public health measures, diagnostic pathways, hospital admission criteria, and therapeutic strategies had already evolved rapidly, complicating the interpretation of sex-related biological effects (4).

The earliest phase of the pandemic in Italy provides an opportunity to isolate intrinsic host determinants of disease expression. During late February and early March 2020, viral circulation was uncontrolled, personal protective equipment was largely unavailable, and no vaccines or disease-specific therapies existed (4). Hospital admissions included a broad, unselected population, and diagnosis frequently relied on clinical and radiological features due to limited RT-PCR testing capacity and suboptimal test sensitivity (5). Chest computed tomography (CT) rapidly emerged not only as a diagnostic tool but also as a robust marker of the extent of lung injury, providing prognostic information on disease severity, hypoxemia, and clinical outcomes, demonstrating high sensitivity for interstitial pneumonia compatible with SARS-CoV-2 infection, even in RT-PCR-negative patients (6).

Sex-related differences in immune responses are well established across infectious diseases. Females typically develop stronger innate and adaptive immune responses, which may enhance viral clearance while limiting excessive inflammatory activation (7). Potential mechanisms include X-linked immune regulatory gene expression, hormonal influences such as estrogen-mediated modulation of cytokine signaling, and differential angiotensin-converting enzyme 2 (ACE2) expression and activity (8). These pathways may influence viral entry, replication, endothelial dysfunction, and thrombo-inflammatory responses, ultimately affecting pulmonary and systemic manifestations. These sex-related differences may be particularly relevant at the level of the lung, where alveolar-capillary injury, endothelial dysfunction, and dysregulated inflammatory responses play a central role in the development of hypoxemia and respiratory failure in COVID-19.

Despite this mechanistic framework, clinical studies addressing sex differences in COVID-19 have produced heterogeneous findings, often influenced by confounders such as differing exposure patterns, viral variants, vaccination status, and evolving therapeutic protocols (3, 9). Furthermore, outcomes may have been modified by the later introduction of corticosteroids, anticoagulation strategies, and antiviral therapies (10). Therefore, early real-world cohorts without these confounding elements are essential to evaluate intrinsic sex-related disease vulnerability.

Addressing this gap in the literature, the present study analyzes a cohort of 689 consecutive patients hospitalized at Parma University Hospital during the pre-lockdown phase in Italy (28 February−22 March 2020) to investigate sex-based differences in clinical presentation, radiological involvement, laboratory profiles, and in-hospital outcomes during the initial COVID-19 outbreak.

## Materials and methods

This retrospective observational study was conducted at Parma University Hospital, a tertiary-care academic center designated as the primary hub for COVID-19 management during the earliest phase of the pandemic in the Parma province (approximately 450,000 inhabitants). The study period extended from 28 February to 22 March 2020, corresponding to the pre-lockdown phase in Italy. During this interval, viral circulation was uncontrolled, personal protective equipment was not yet routinely available, and no vaccines or disease-specific treatments existed. Hospital admission criteria were broad and based primarily on clinical presentation and radiological suspicion of COVID-19 pneumonia, with nasopharyngeal swab testing (RT-PCR) also used to confirm the diagnosis. Consecutive adult patients (≥18 years) admitted with suspected COVID-19 infection and chest computed tomography (CT) showing findings consistent with interstitial pneumonia were included in the analysis. A total of 689 patients met these criteria. COVID-19 infection was confirmed by reverse transcription polymerase chain reaction (RT-PCR) when available; however, patients with negative RT-PCR swabs were retained if CT imaging demonstrated patterns typical of COVID-19 pneumonia. No exclusion criteria were applied on the basis of comorbidities, frailty, or treatment limitations. Patients with incomplete core clinical or radiological data were excluded. Demographic data, comorbidities, chronic medication use, clinical symptoms, time from symptom onset to hospital presentation, laboratory findings at admission, arterial blood gas results, and radiological characteristics were extracted from electronic medical records using a standardized template. Frailty was assessed using the Rockwood Clinical Frailty Scale, based on pre-admission functional status as documented in medical records and caregiver reports, according to standard definitions. Comorbidity burden was expressed as the total number of pre-existing chronic conditions. Chest CT scans were interpreted by experienced radiologists. Disease extent was quantified using a semi-quantitative CT visual score reflecting the estimated percentage of lung parenchyma involvement. The presence of ground-glass opacities and consolidations was recorded. The CT visual score was analyzed both as a descriptive variable and as a predictor in multivariable regression models. In RT-PCR–negative patients, COVID-19 diagnosis was supported by chest CT patterns considered typical for SARS-CoV-2 infection at that time, including bilateral ground-glass opacities with peripheral and posterior distribution, with or without consolidations, in the appropriate clinical context. Chest CT scans were independently reviewed by experienced radiologists involved in routine COVID-19 care during the study period. Radiologists were blinded to clinical outcomes. Lung involvement was visually estimated as the percentage of affected parenchyma and expressed as a semi-quantitative CT visual score. Discrepancies were resolved by consensus. No patients were excluded from the analysis because of missing core clinical or radiological data.

The primary outcome was in-hospital mortality. Secondary clinical outcomes included oxygen saturation levels, PaO2/FiO2 ratio, maximum oxygen flow requirement, need for non-invasive ventilation (NIV), transfer to intensive care unit (ICU), and length of hospital stay. Outcomes were compared between males and females.

### Statistical analysis

Continuous variables were reported as median and interquartile range (IQR), and comparisons were performed using the Mann–Whitney *U*-test. Categorical variables were expressed as percentages and compared using the Chi-square test. Age-adjusted analyses were conducted using Quade's non-parametric ANCOVA for continuous variables and binary logistic regression for categorical variables. Associations between CT visual score and laboratory or clinical variables were examined using multivariable linear regression with stepwise selection. A separate multivariable stepwise linear regression was used to identify factors associated with PaO2/FiO2 at admission. In-hospital mortality was analyzed using multivariable logistic regression. Odds ratios (OR) with 95% confidence intervals (CI) were calculated. A two-sided *p*-value < 0.05 was considered statistically significant. To further assess sex-related differences in survival over time, a multivariable Cox proportional hazards regression model was also performed, including age, CT visual score, PaO_2_/FiO_2_, number of chronic illnesses, RT-PCR positivity, and sex. Statistical analyses were performed using SPSS software (version 29.0; IBM, Armonk, NY, USA).

### Ethical approval

Ethics Committee approval was obtained (Comitato Etico dell'Area Vasta Emilia Nord, Emilia-Romagna region) under the ID 273/2020/OSS/AOUPR as part of a larger project on the characteristics of patients hospitalized with confirmed or suspected COVID-19 during the first pandemic wave. All participants, who were contactable by phone or for follow-up reasons, provided written informed consent for participation. For all other cases, the Ethics Committee, in accordance with the guidelines in force at the moment of approval, waived written informed-consent collection due to the retrospective design of the study.

## Results

A total of 689 patients were hospitalized during the study period, of whom 422 (61%) were male and 267 (39%) were female. The median age was 71 years (IQR 60–80), with females being significantly older than males (73 vs. 70 years). Frailty was more pronounced in females, with a higher prevalence of Rockwood ≥7 (15 vs. 5%; *p* < 0.001), while the number of chronic comorbidities was comparable between sexes. However, specific conditions differed: chronic heart disease, diabetes, dyslipidemia, and COPD were more frequent in males, whereas thyroid disorders, dementia, epilepsy, and malignancy were more common in females. Clinical presentation was often non-specific. Fever was reported more frequently in males (91 vs. 84%; *p* = 0.003), whereas atypical symptoms and asthenia were more common among females (19 vs. 14% and 13 vs. 8%, respectively; Table 1).

**Table 1:** Patients hospitalized between 28 February and 22 March 2020 with CT-Confirmed ground-glass opacities, stratified by sex.

**N.689**	**Males *N*.422**	**Females *N*.267**	***p***	^*^ *P*
Age, years	70 (58–78)	73 (63–82)	**< 0.001**	
Rockwood ≤ 4, %	77	68	**0.012**	0.914
Rockwood ≥7, %	5	15	**< 0.001**	**0.003**
Chronic comorbidities, number	2 (1–4)	2 (1–4)	0.133	0.688
Systemic drugs, number	3 (1–5)	3 (1–6)	**0.030**	0.829
Consolidations on chest CT, %	68	75	0.058	0.069
RT-PCR positive on admission, %	76	75	0.754	0.529
Anamnestic data				
CHA_2_DS_2_Vasc score	2 (1–3)	3 (2–4)	**< 0.001**	**< 0.001**
Hypertension, %	56	58	0.650	0.404
Diabetes, %	22	17	0.160	**0.046**
Obesity, %	14	10	0.068	0.120
Dyslipidemia, %	19	14	0.091	**0.031**
Chronic heart disease, %	22	18	0.219	**0.007**
Arrhythmia, %	10	17	**0.006**	0.209
COPD, %	11	6	**0.022**	**0.003**
Cancer, %	10	16	**0.013**	**0.026**
Dementia, %	5	13	**< 0.001**	**0.025**
Vascular disease, %	8	7	0.519	0.120
Thyroid disorders, %	7	22	**< 0.001**	**< 0.001**
Epilepsy, %	1	4	**0.006**	**0.009**
Antipsychotics, %	3	7	**0.018**	0.091
Antidepressants, %	9	18	**< 0.001**	**0.003**
Antiepileptics, %	3	6	**0.049**	0.050
Antiplatelet drugs, %	29	25	0.253	**0.011**
Corticosteroids, %	3	6	0.071	0.139
OAC/NOAC, %	8	13	0.058	0.578
Diuretics, %	22	30	**0.018**	0.275
Clinical presentation of suspect COVID-19				
Symptom duration, days	7 (5–10)	7 (3–10)	**0.014**	**0.006**
Cough, %	56	49	0.067	0.238
Fever, %	91	84	**0.003**	**0.028**
Dyspnea, %	47	46	0.815	0.432
Diarrhea, %	7	6	0.448	0.556
Asthenia, %	8	13	**0.023**	**0.014**
Atypical symptoms, %	14	19	**0.047**	**0.049**
Parameters on admission				
CT visual score, %	30 (20–50)	25 (15–40)	**< 0.001**	**< 0.001**
Temperature on admission, °C	37.1 (36.0–37.8)	36.8 (36.0–37.5)	**0.013**	**0.026**
O_2_ saturation on admission, %	95 (92–97)	95 (93–97)	**0.005**	**0.001**
Arterial blood gas analysis				
pH	7.45 (7.43–7.48)	7.45 (7.42–7.48)	0.327	0.478
${\text{HC)}}_{3}^{-}$, mmol/L	25 (23–27)	25 (23–28)	0.088	**0.036**
pCO_2_, mmHg	36 (32–39)	36 (33–39)	**0.008**	**0.003**
pO_2_, mmHg	70 (58–83)	72 (61–89)	**0.032**	**0.010**
pO_2_/FiO_2_	240 (121–324)	240 (147–338)	0.205	**0.032**

Data are shown as median and interquartile range or percentages.

*p* calculated with Mann–Whitney test or chi-square test, as appropriate.

p^*^ adjusted for age with Quade's non-parametric ANCOVA or binary logistic regression.

*p*-values < 0.05 are indicated in bold.

Laboratory findings reflected more severe systemic involvement in males. Higher levels of C-reactive protein, LDH, creatinine, and CPK were observed, along with a higher prevalence of lymphopenia (61 vs. 53%). In contrast, platelet counts and normal procalcitonin values were more frequent in females (Table 2).

**Table 2:** Laboratory parameters on admission by sex.

**Parameter**	**Males N.422**	**Females N.267**	***p***	*P* ^*^
**Clinical chemistry and hematology**				
Hemoglobin, g/dl	14.2 (13.2–15.2)	13.0 (11.8–14.1)	**< 0.001**	**< 0.001**
Platelets, 1,000/mm^3^	196 (158–246)	211 (168–277)	**0.017**	**0.007**
White blood cells, n/mm^3^	6,680 (5,035–8,965)	6,020 (4,400–8,830)	**0.024**	**0.007**
Neutrophils, n/mm^3^	5,103 (3,678–7,474)	4,550 (2,964–6,986)	**0.015**	**0.003**
Lymphocytes, n/mm^3^	893 (630–1,196)	982 (652–1,300)	**0.039**	**0.005**
Creatinine, mg/dl	1.00 (0.80–1.20)	0.70 (0.60–1.00)	**< 0.001**	**< 0.001**
Urea, mg/dl	46 (35–63)	38 (28–61)	**< 0.001**	**< 0.001**
AST, UI/L	52 (36–77)	43 (32–62)	<**0.001**	<**0.001**
CPK, UI/L	177 (101–424)	112 (67–225)	**< 0.001**	**< 0.001**
LDH, UI/L	371 (292–500)	337 (269–449)	**0.004**	**0.001**
D-Dimer, ng/dl	992 (639–1,749)	954 (618–1,421)	0.216	**0.047**
Fibrinogen, ng/dl	647 (536–779)	580 (469–708)	**< 0.001**	**< 0.001**
C-reactive protein, mg/L,	112 (60–179)	77 (39–135)	**< 0.001**	**< 0.001**
Procalcitonin, ng/ml	0.19 (0.10–0.53)	0.12 (0.06–0.29)	**< 0.001**	**< 0.001**
INR ratio	1.21 (1.16–1.30)	1.18 (1.10–1.29)	**0.001**	**0.001**
PT	1.21 (1.15–1.30)	1.18 (1.11–1.28)	**< 0.001**	**< 0.001**
aPTT ratio	0.98 (0.91–1.06)	0.94 (0.88–1.04)	**0.002**	**0.001**
Lymphocytes < 1,000/mm^3^, %	61	53	**0.033**	**0.008**
C-reactive protein ≥5 and < 40 mg/L, %	14	21	**0.012**	**0.003**
C-reactive protein ≥40 and < 200 mg/L, %	63	64	0.726	0.978
C-reactive protein ≥200 mg/L, %	21	10	**< 0.001**	**< 0.001**
Procalcitonin < 0.05 ng/ml, %	7	16	**0.001**	**< 0.001**
Procalcitonin ≥0.05 and < 0.5 ng/ml, %	67	68	0.805	0.893
Procalcitonin ≥0.5, **%**	26	17	**0.009**	**0.001**

Data are shown as median and interquartile range or percentages.

*p* calculated with Mann–Whitney test or chi-square test, as appropriate.

p^*^ adjusted for age with Quade's non-parametric ANCOVA or binary logistic regression.

*p*-values < 0.05 are indicated in bold.

AST, aspartate aminotransferase; CPK, creatine-phosphokinase; LDH, lactate-dehydrogenase.

**Figure 1:**
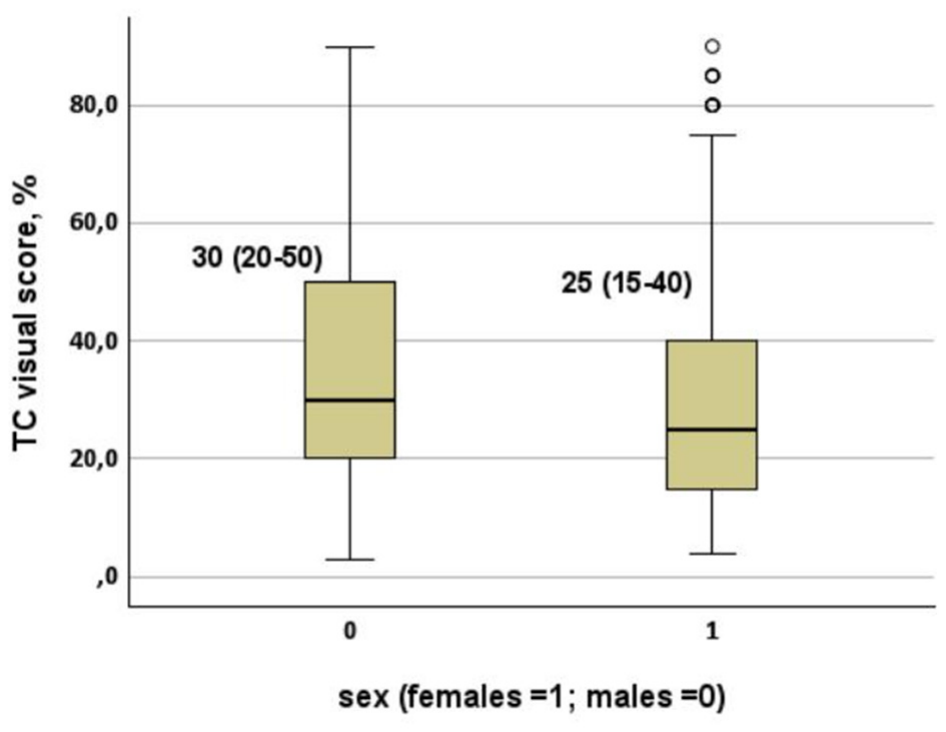
CT visual score (whiskers plots) in patients hospitalized between 28 February and 22 March 2020 for COVID-19 pneumonia stratified by sex (*p* < 0.001). Distribution of CT visual score according to sex (whisker plots). Females showed significantly lower lung involvement than males (*p* < 0.001).

Radiological severity mirrored these differences. The median CT visual score was significantly lower in females (25 vs. 30%; *p* < 0.001), indicating reduced pulmonary involvement (Table 1). This difference was clearly evident in the distribution of CT visual scores, which showed a marked leftward shift in females (Figure 1). Multivariable regression identified LDH, D-dimer, C-reactive protein, and platelet count as independent predictors of visual score severity (Table 3), while PaO_2_/FiO_2_ impairment was primarily associated with visual score, inflammatory markers, comorbidity burden, and age (Table 4).

**Table 3:** Determinants of CT visual score extent identified by stepwise linear regression.

**Variable**	**Standardized beta**	***p***
LDH, UI/L	0.460	< 0.001
D-Dimer, ng/dl	0.216	< 0.001
C-reactive protein, mg/L	0.209	< 0.001
Platelets, 1,000/mm^3^	0.126	0.001

R = 0.717, R^2^ = 0.515, *p* < 0.001.

This table shows the independent variables associated with the extent of the CT visual score, as identified through stepwise linear regression analysis.

Standardized beta coefficients and corresponding *p*-values are reported.

The final model demonstrated good explanatory capacity (R = 0.717; R^2^ = 0.515; p < 0.001), indicating that LDH, D-dimer, C-reactive protein, and platelet count significantly contributed to the variability of the visual score.

**Table 4:** Factors associated with admission PaO_2_/FiO_2_ identified through stepwise linear regression.

**Variable**	**Standardized beta**	***p***
CT visual score, %	−0.297	< 0.001
C-reactive protein, mg/L	−0.205	< 0.001
Chronic comorbidities, number	−0.184	< 0.001
LDH, UI/L	−0.152	0.001
Dyspnea, %	−0.124	0.006
Age, years	−0.106	0.030
Hemoglobin, g/dl	−0.091	0.045

R = 0.689, R^2^ = 0.475, *p* < 0.001.

This table presents the factors independently associated with PaO_2_/FiO_2_ ratio at hospital admission.

Negative standardized beta coefficients indicate variables associated with lower oxygenation.

The model (R = 0.689; R^2^ = 0.475; *p* < 0.001) highlights the influence of CT visual score, inflammatory biomarkers, comorbidity burden, LDH levels, dyspnea, age, and hemoglobin on respiratory impairment at presentation.

Clinical course indicators were consistently more favorable in females. They required lower maximum oxygen flow rates and experienced shorter hospital stays. Non-invasive ventilation and ICU admission were comparable between sexes (Table 5).

**Table 5:** Clinical course in patients stratified by sex.

**Parameter**	**Males N.422**	**Females N.267**	***p***	*P* ^*^
Worst O_2_ saturation during stay, %	90 (84–93)	91 (86–94)	**0.008**	**< 0.001**
Maximum O_2_ flows during stay, %	50 (30–75)	40 (28–75)	**0.043**	**0.001**
Worst arterial O_2_ pressure, mmHg	56 (44–66)	59 (48–71)	**0.005**	**0.001**
Temperature peak during stay, °C	38.2 (37.3–38.8)	38.0 (37.1–38.6)	**0.003**	**0.011**
Length of hospitalization, days	6 (3–12)	5 (2–10)	**0.029**	**0.045**
Non-invasive ventilation during stay, %	14	9	**0.049**	0.108
Intensive care unit, %	6	4	0.207	0.336
Death, %	28	25	0.515	**0.009**

Data are shown as median and interquartile range or percentages.

*p* calculated with Mann–Whitney test or chi-square test, as appropriate.

p^*^ adjusted for age with Quade's non-parametric ANCOVA or binary logistic regression.

*p*-values < 0.05 are indicated in bold.

**Figure 2:**
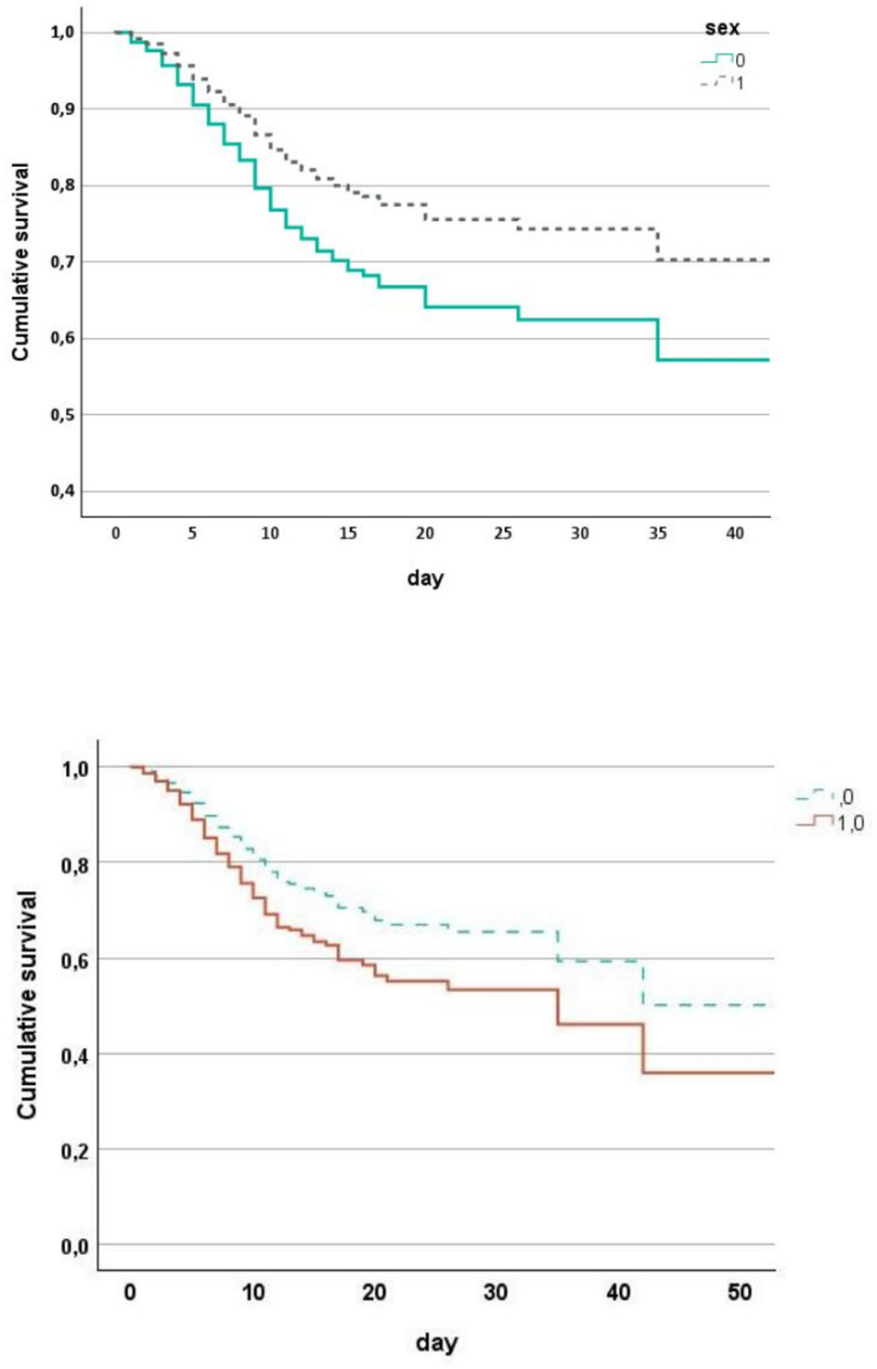
Cumulative survival in patients hospitalized between 28 February and 22 March 2020 for COVID-19 pneumonia stratified by sex (females = 1 and males = 0) after adjustment for age, CT visual score, pO_2_/FiO_2_, chronic illnesses, RT-PCR positive on admission. Adjusted Kaplan–Meier survival curves according to sex, controlling for age, CT visual score, PaO_2_/FiO_2_, number of chronic illnesses, and RT-PCR positivity.

Overall mortality was 28% in males and 25% in females, but after age adjustment the difference became significant, confirming lower mortality in females (Table 6). Kaplan–Meier survival analysis adjusted for age, CT visual score, PaO_2_/FiO_2_, comorbidity burden, and RT-PCR positivity confirmed a significantly better survival in females compared with males (Figure 2). Multivariable logistic regression demonstrated that age, comorbidity burden, and RT-PCR positivity were independently associated with mortality, while female sex was protective (OR 0.597; Table 7). In time-to-event analysis, a multivariable Cox proportional hazards model confirmed the independent association between female sex and lower in-hospital mortality (hazard ratio 0.63, *p* = 0.012), together with age, CT visual score, PaO_2_/FiO_2_, comorbidity burden, and RT-PCR positivity (Table 8).

**Table 6:** Outcomes by sex and test result.

**Outcome**	**RT-PCR negative on admission**				**RT-PCR positive on admission**			
	**Males N.103**	**Females N.68**	***p***	*P* ^*^	**Males N.319**	**Females N.199**	***p***	*P* ^*^
Age, years	67 (54–74)	71 (61–82)	**0.023**		71 (60–79)	75 (63–83)	**0.002**	
Non-invasive ventilation during stay, %	13	9	0.448	0.462	14	9	0.069	0.148
Intensive care unit, %	5	6	0.770	0.673	7	4	0.104	0.184
Death, %	15	12	0.602	0.105	32	30	0.664	**0.045**

Data are shown as median and interquartile range or percentages.

*P* for age calculated with Mann–Whitney test.

*p* and p^*^ for percentages calculated with binary logistic regression without and with age adjustment.

*p*-values < 0.05 are indicated in bold.

**Table 7:** Association of age, chronic illnesses, RT-PCR positivity, and sex with hospital mortality.

**Variable**	**Odds ratio**	**95% confidence interval**	***p***
Age, years	1.082	1.061–1.104	< 0.001
Chronic illnesses, number	1.256	1.133–1.392	< 0.001
RT-PCR positive on admission	2.637	1.556–4.469	< 0.001
Females	0.597	0.396–0.898	0.013

This table summarizes the univariate logistic regression analysis evaluating the association of demographic and clinical characteristics with hospital mortality.

Odds ratios with 95% confidence intervals and *p*-values are provided.

Older age, a higher number of chronic illnesses, and RT-PCR positivity at admission were associated with increased mortality risk, whereas female sex was associated with reduced mortality.

**Table 8:** Risk of death in hospital in patients with COVID-19 pneumonia tested with Cox regression multivariate analysis.

**Variable**	**Hazard ratio**	**95% CI for hazard ratio**	***p***
Age, years	1.073	1.053–1.093	< 0.001
CT visual score, %	1.016	1.008–1.024	< 0.001
pO_2_/FiO_2_	0.995	0.993–0.998	< 0.001
Chronic illnesses, number	1.122	1.029–1.224	0.009
RT-PCR positive on admission	2.029	1.225–3.359	0.006
Females	0.631	0.440–0.904	0.012

This table displays the results of the multivariate stepwise logistic regression model assessing independent predictors of hospital mortality.

Odds ratios, 95% confidence intervals, and *p*-values are reported.

Age, comorbidity burden, RT-PCR positivity, platelet count, PaO_2_/FiO_2_ ratio, and CT visual score emerged as significant contributors to mortality risk.

Despite being significantly older and frailer at admission, females exhibited less extensive radiological lung involvement, better oxygenation, and lower age-adjusted in-hospital mortality compared with males.

## Discussion

In this pre-lockdown cohort of hospitalized patients with radiologically confirmed COVID-19 pneumonia, we observed striking sex-related differences in clinical presentation, inflammatory profile, radiological severity, and outcomes. Females represented a minority of admissions, yet they were significantly older and frailer than males, with a threefold higher prevalence of severe frailty. Based on established prognostic indicators in COVID-19, including advanced age, multimorbidity, and functional impairment, this group would have been expected to experience substantially worse outcomes (11, 12). Instead, females demonstrated a markedly more favorable clinical trajectory, characterized by reduced radiological lung involvement, better oxygenation, lower inflammatory marker levels, shorter hospital stay, and significantly lower age-adjusted mortality.

These findings strongly support the hypothesis that intrinsic sex-related biological mechanisms modulate disease severity in SARS-CoV-2 infection. Several studies have demonstrated higher mortality among males across different countries and clinical settings, independent of demographic structure or healthcare system differences (1, 2). Proposed mechanisms include sex-based variation in immune responses, hormonal regulation, and differences in angiotensin-converting enzyme 2 (ACE2) expression and activity. ACE2, the primary entry receptor for SARS-CoV-2, is expressed differently in males and females, potentially contributing to variation in viral entry and replication dynamics (8). Moreover, estrogens may exert protective effects by modulating cytokine production, enhancing antiviral immune responses, and stabilizing endothelial function (13).

The inflammatory profile observed in our cohort supports these biological considerations. Males displayed higher levels of C-reactive protein, lactate dehydrogenase, and creatine phosphokinase, along with more pronounced lymphopenia, features consistently associated with severe COVID-19 and poor outcomes (14). In contrast, females showed more preserved laboratory parameters despite greater frailty. This suggests that females may mount a more balanced immune response, capable of controlling viral replication while limiting excessive systemic inflammation, a phenomenon previously described in other viral respiratory infections (7).

Radiological findings reinforce this interpretation. The CT visual score, an established marker of pulmonary involvement and disease severity, was significantly lower in females. In multivariable analysis, radiological extent was strongly associated with inflammatory and coagulation markers, supporting the concept that lung involvement reflects systemic inflammatory activity. Importantly, when CT visual score and oxygenation indices were included in regression models, sex lost statistical significance as an independent predictor of mortality, indicating that the survival advantage of females is mediated primarily through reduced pulmonary damage and better respiratory physiology (Table 8).

Taken together, these findings support a lung-centered interpretation of sex-related differences in COVID-19 severity, whereby females appear relatively protected from extensive alveolar-capillary damage, resulting in preserved gas exchange and reduced progression toward hypoxemic respiratory failure. The graphical representation of lung involvement and survival further supports this interpretation. The whisker plot of CT visual score demonstrates a clear separation between sexes, with females clustering at lower levels of radiological lung injury (Figure 1), while adjusted Kaplan–Meier curves confirm a persistent survival advantage in women over time (Figure 2).

The early pandemic context of this cohort provides a unique perspective. Unlike later phases characterized by reduced viral circulation, widespread mask use, vaccination campaigns, and evolving treatment strategies, patients in this study were exposed to high viral loads and received no specific antiviral or immunomodulatory therapies. Therefore, observed sex differences are unlikely to be influenced by differential access to care, treatment response, or vaccination status. Similar observations were reported in early cohorts from China and the United States, where males consistently showed higher rates of severe disease and mortality before treatment standardization (15, 16).

The high proportion of RT-PCR-negative patients with typical CT patterns highlights limitations of early diagnostic testing and underscores the importance of radiological assessment during this phase. Interestingly, mortality differences between sexes were most evident among RT-PCR-positive patients, suggesting that viral burden may interact with sex-related biological factors. Previous studies have reported that viral load, measured by cycle threshold values, was higher in males and associated with worse outcomes (17), potentially contributing to the observed differences.

Our findings also align with literature exploring the interaction between frailty and COVID-19 outcomes. Frailty is a recognized predictor of mortality and functional decline in hospitalized older adults (18, 19). However, in our cohort, frailty did not attenuate the protective effect of female sex, suggesting that biological resilience may persist even in highly vulnerable individuals.

This study has several strengths. The cohort represents a real-world, unselected population hospitalized during the earliest phase of the pandemic, allowing the assessment of intrinsic host factors with minimal confounding from treatments or varying exposure patterns. Radiological confirmation of pneumonia ensured diagnostic accuracy even in RT-PCR negative cases. Furthermore, the availability of detailed laboratory and clinical data enabled robust multivariable analyses.

However, limitations must be acknowledged. The retrospective design may introduce information bias, although data extraction was standardized. Frailty assessment relied on documented clinical information rather than direct evaluation. Viral load measurements were not available, preventing direct correlation between sex, viral burden, and outcomes. Although no standardized COVID-19-specific therapies were available during the study period, minor sex-related differences in supportive treatments cannot be entirely excluded and may have partially influenced clinical trajectories. Finally, the absence of long-term follow-up limits evaluation of post-acute sequelae.

Overall, this study provides compelling evidence that females exhibit a more favorable response to SARS-CoV-2 infection during the early pandemic phase, despite older age and higher frailty. The protective effect appears largely mediated by reduced pulmonary involvement and attenuated inflammatory response. Understanding the biological basis of this resilience may inform personalized therapeutic strategies and improve risk stratification in future respiratory pandemics.

## Conclusions

In this large pre-lockdown cohort of hospitalized patients with radiologically confirmed COVID-19 pneumonia, we identified clear and clinically meaningful sex-related differences in disease expression and outcomes. Despite being significantly older and exhibiting higher levels of frailty, females presented with less extensive pulmonary involvement, more favorable respiratory parameters, and a markedly attenuated inflammatory and tissue-damage profile. These characteristics translated into shorter hospital stays, reduced oxygen requirements, and significantly lower age-adjusted mortality compared with males.

The particular context of this cohort, characterized by high viral exposure, absence of vaccination, and lack of disease-specific treatments, provides valuable insight into the intrinsic biological response to SARS-CoV-2 infection.

These results contribute to the understanding of COVID-19 pathophysiology by demonstrating that female resilience is evident even in the presence of advanced age and frailty, factors traditionally associated with poorer prognoses. Recognizing sex-specific patterns in disease manifestation may support more accurate risk stratification and inform personalized clinical management strategies in future respiratory pandemics.

## Data Availability

The raw data supporting the conclusions of this article will be made available by the authors, without undue reservation.
